# Direct comparison of Xpert Xpress, FilmArray Respiratory Panel, Lumipulse antigen test, and RT-qPCR in 165 nasopharyngeal swabs

**DOI:** 10.1186/s12879-022-07185-w

**Published:** 2022-03-04

**Authors:** Yosuke Hirotsu, Makoto Maejima, Masahiro Shibusawa, Yume Natori, Yuki Nagakubo, Kazuhiro Hosaka, Hitomi Sueki, Kenji Amemiya, Miyoko Hayakawa, Hitoshi Mochizuki, Toshiharu Tsutsui, Yumiko Kakizaki, Yoshihiro Miyashita, Masao Omata

**Affiliations:** 1 Genome Analysis Center, Yamanashi Central Hospital, 1-1-1 Fujimi, Kofu, Yamanashi Japan; 2 Division of Microbiology in Clinical Laboratory, Yamanashi Central Hospital, 1-1-1 Fujimi, Kofu, Yamanashi Japan; 3 Division of Genetics and Clinical Laboratory, Yamanashi Central Hospital, 1-1-1 Fujimi, Kofu, Yamanashi Japan; 4 Central Clinical Laboratory, Yamanashi Central Hospital, 1-1-1 Fujimi, Kofu, Yamanashi Japan; 5 Department of Gastroenterology, Yamanashi Central Hospital, 1-1-1 Fujimi, Kofu, Yamanashi Japan; 6 Lung Cancer and Respiratory Disease Center, Yamanashi Central Hospital, 1-1-1 Fujimi, Kofu, Yamanashi Japan; 7 The University of Tokyo, 7-3-1 Hongo, Bunkyo-ku, Tokyo, Japan

**Keywords:** SARS-CoV-2, COVID-19, Xpert Xpress, FilmArray, Lumipulse

## Abstract

**Background:**

The nucleic acid amplification test (NAAT) and antigen test are approved diagnostic tests for COVID-19. In this study, we aimed to investigate the assay performance of two NAATs (Xpert Xpress SARS-CoV-2 and FilmArray Respiratory Panel) and a quantitative antigen test (Lumipulse).

**Methods:**

One hundred and sixty-five nasopharyngeal swabs were subjected to Xpert, FilmArray, Lumipulse, and RT-qPCR assays.

**Results:**

Of 165 samples, RT-qPCR showed 100 positives and 65 negatives. The Xpert had an overall agreement of 99.4% (95% confidence interval [CI]: 96.7–99.4%), sensitivity of 99% (95% CI: 96.8–99%), and specificity of 100% (95% CI: 96.6–100%). FilmArray had an overall agreement of 98.8% (95% CI: 95.9–98.8%), sensitivity of 98% (95% CI: 95.6–98%), and specificity of 100% (95% CI: 96.3–100%). Lumipulse had an overall agreement of 95.5% (95% CI: 91.8–95.5%), sensitivity of 92.3% (95% CI: 89.2–92.3%), and specificity of 100% (95% CI: 95.5–100%). The κ coefficient showed excellent agreement between each test and RT-qPCR. There was a high correlation between Xpert Ct values, RT-qPCR Ct values, viral loads and antigen level.

**Conclusions:**

Xpert Xpress and FilmArray Respiratory Panel exhibited an equivalent performance. The Lumipulse antigen test was slightly less sensitive than the NAATs, but showed high assay performance except for samples with low viral load. The Xpert Xpress, FilmArray Respiratory Panel and Lumipulse antigen tests offer rapid sample-to-answer data, allowing random access detection on automated devices.

## Background

The emergence of novel Variants of Concern (VOCs) is threatening human life [1–5]. In particular, the spread of the Alpha, Beta, Gamma, Delta and Omicron variant has led to an infection outbreak in several countries [6–8]. Therefore, diagnostic tests for coronavirus disease 2019 (COVID-19) continue to be in high demand.

Reverse transcription-polymerase chain reaction (RT-PCR) is the standard method for detecting severe acute respiratory syndrome coronavirus 2 (SARS-CoV-2) [9]. The steps of RT-PCR are as follows: extract genomic RNA from the virus, reverse transcribe into complementary DNA, amplify the region of interest, and detect fluorescent signals. This method amplifies and detects viral-derived nucleic acids, making it possible to sensitively evaluate samples with low viral loads. However, when performed manually, it requires skilled techniques and hands-on time, taking approximately 3–4 h to obtain the results.

Automated devices for testing SARS-CoV-2 have been developed. Random access detection with automated devices enables the rapid return of results to clinicians and patients. In addition, once the sample is loaded into the dedicated reagent, the process from sample to answer is seamless. These tests are straightforward to perform and do not always require skilled personnel. In addition, random access detection can be performed for each sample, making it useful for emergency testing.

We previously showed that the FilmArray Respiratory Panel (v2.1) and Lumipulse antigen test had high accuracy for detecting SARS-CoV-2 in nasopharyngeal swab samples [10–12]. Next, we planned to strengthen our testing system for SARS-CoV-2 by installing Xpert Xpress SARS-CoV-2. Previous studies reported the performance of Xpert compared with that of the Cobas SARS-CoV-2 assay or in-house real-time RT-PCR assays using clinical samples [13–17]. However, there have been no reports of a head-to-head comparison between the Xpert and FilmArray or Lumipulse. In this study, we aimed to evaluate the assay performance of the Xpert Xpress SARS-CoV-2 and FilmArray Respiratory Panel as automated RT-PCR assays and tested the Lumipulse as an automated antigen quantification test. The same samples were also tested for quantitative RT-PCR (RT-qPCR) as the reference, and the results were compared.

## Methods

### Patients and samples

A total of 165 nasopharyngeal swabs were collected and stored in viral transport medium (VTM) (Copan, Murrieta, CA, USA). The patients comprised 78 women and 87 men, with a mean age of 49.2 years (range 1–94). The Institutional Review Board at Yamanashi Central Hospital approved this study and the use of an opt-out consent method (G-2019-1). The requirement for written informed consent was waived because this was an observational study. Patient participation in the study was optional.

### Viral nucleic acid extraction

We extracted total nucleic acid from 200 μL of VTM using a MagMax Viral/Pathogen Nucleic Acid Isolation Kit (Thermo Fisher Scientific, Waltham, MA, USA) on a KingFisher Duo Prime (Thermo Fisher Scientific) as previously described [18, 19]. These extracted nucleic acids were analyzed by quantitative reverse transcription PCR (RT-qPCR). The residual VTM was used for testing with the Xpert Xpress SARS-CoV-2, FilmArray Respiratory Panel, and Lumipulse SARS-CoV-2 antigen test as described below.

### RT-qPCR

To detect SARS-CoV-2, we performed one-step RT-qPCR in accordance with the protocol developed by the National Institute of Infectious Diseases in Japan [20]. This method amplifies the nucleocapsid (*N*) gene of SARS-CoV-2 (NC_045512.2) [9]. We made reaction mixture composed of 5 µL of 4 × TaqMan Fast Virus 1-Step Master Mix (Thermo Fisher Scientific), 1.0 μL of 10 μM forward primer, 1.4 μL of 10 μM reverse primer, 0.8 μL of 5 μM probe, 6.8 µL of nuclease-free water, and 5 µL of the nucleic acid sample in a 20-μL total volume as previously described [9].

The StepOnePlus Real-Time PCR System (Thermo Fisher Scientific) was used for RT-qPCR assays as following condition: 50 °C for 5 min, 95 °C for 20 s, and 45 cycles of 95 °C for 3 s and 60 °C for 30 s. The threshold cycle was examined then the threshold was set at 0.2. We judged the results according to the protocol (version 2.9.1) [20].

### Xpert Xpress SARS-CoV-2

The Xpert Xpress SARS-CoV-2 (Cepheid, Sunnyvale, CA) is an automated real-time RT-PCR test carried out in single-use disposable cartridges. For this assay, 300 µL of VTM was loaded into the sample chamber of the cartridge using a sterile pipette. The cartridges were set in a GeneXpert System (Cepheid) and analyzed. This assay targets the envelope (*E*) gene and *N* gene (named N2) and detects SARS-CoV-2 according to the cycle threshold cut-off values.

### FilmArray Respiratory Panel

We performed multiplex real-time RT-PCR assay using the FilmArray Respiratory Panel version 2.1 (bioMérieux, Marcy-l’Etoile, France) [21]. This assay detects 21 pathogens (i.e., SARS-CoV-2 and 17 other viruses and three bacteria) related to respiratory diseases. The kit buffer and 300 µL of VTM were injected into the FilmArray pouch and the reaction proceeded on the FilmArray Torch system (bioMérieux).

### Lumipulse SARS-CoV-2 antigen test

The SARS-CoV-2 antigen levels were determined quantitatively with the Lumipulse SARS-CoV-2 antigen test (Fujirebio, Inc., Tokyo, Japan) as previously described [12]. Briefly, 700 μL of the VTM were vortexed and centrifuged at 2000×*g* for 5 min. Aliquots (100 μL) of the supernatant were tested on the LUMIPULSE G600II system (Fujirebio). For samples with an antigen level > 5000 pg/mL, the samples were diluted with the kit diluent and tested again, and the antigen level was calculated based on the dilution factor. We judged the results according to the manufacturer’s instruction.

### Statistical analysis

Sensitivity and specificity values were calculated using the RT-qPCR results as the reference and with the exclusion of inconclusive samples determined by Lumipulse antigen testing. Cohen’s kappa (κ) coefficient of results between the two tests with 95% confidence intervals (CI) was calculated. Cohen’s κ values > 0.81 were interpreted to indicate near-perfect agreement [22]. Statistical analysis was performed using R Version 3.1.2 (http://www.r-project.org/).

## Results

### Test accuracy

**Fig. 1:**
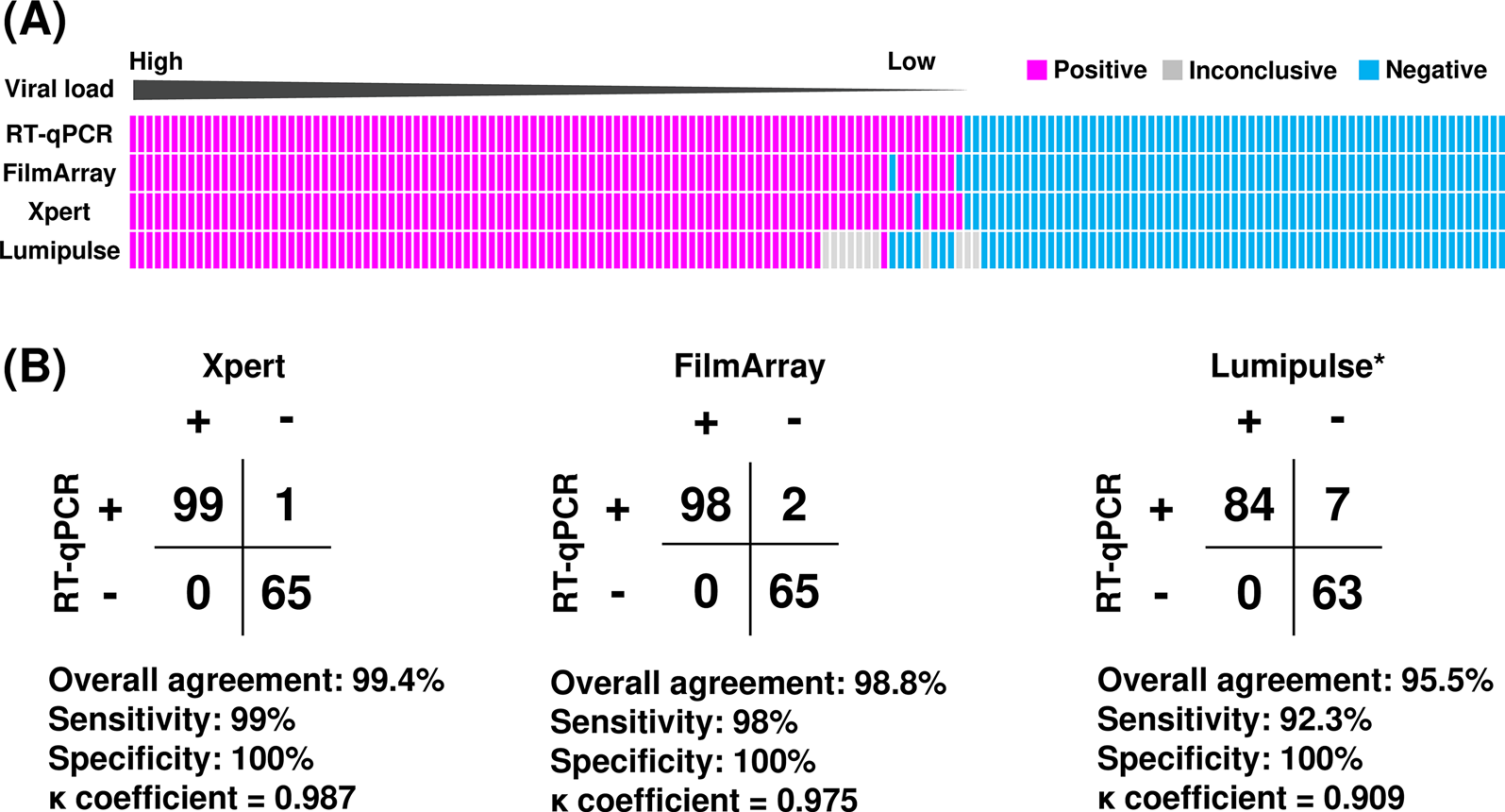
Comparison of RT-qPCR, Xpert, FilmArray, and Lumipulse test results. **A** Analysis of 165 nasopharyngeal swabs using RT-qPCR, with data shown from the highest viral load (left) to the lowest (right). Pink in the box indicates positive, gray indicates inconclusive, and light blue indicates negative. **B** Comparison of Xpert, FilmArray, and Lumipulse with RT-qPCR results as a reference. *The Lumipulse data exclude 11 inconclusive samples. + positive; − negative

A total of 165 nasopharyngeal swab samples were analyzed to investigate the accuracy of test results. Of the 165 samples, 100 were RT-qPCR positive, and 65 were RT-qPCR negative (Fig. 1A). Using these samples, Xpert, FilmArray, and Lumipulse tests were conducted.

When the results of RT-qPCR were used as a reference, the overall agreement rate of Xpert was 99.4% (95% CI: 96.7–99.4%) with a sensitivity of 99% (95% CI: 96.8–99%) and specificity of 100% (95% CI: 96.6–100%) (Fig. 1B). The overall agreement rate of FilmArray was 98.8% (95% CI: 95.9–98.8%) with a sensitivity of 98% (95% CI: 95.6–98%) and specificity of 100% (95% CI: 96.3–100%) (Fig. 1B). When inconclusive results (n = 11) were excluded for the Lumipulse test (Fig. 1A), the overall agreement was 95.5% (95% CI: 91.8–95.5%) with a sensitivity of 92.3% (95% CI: 89.2–92.3%) and specificity of 100% (95% CI: 95.5–100%) (Fig. 1B). The κ coefficient indicated excellent agreement between each test and RT-qPCR (Xpert vs RT-qPCR, κ = 0.987, 95% CI: 0.932–0.987; FilmArray vs RT-qPCR, κ = 0.975, 95% CI: 0.914–0.975; Lumipulse vs RT-qPCR, κ = 0.909, 95% CI: 0.836–0.909). Compared to Lumipulse, Xpert and FilmArray were equivalent in accuracy to RT-qPCR.

### Detectable range and discordant results

**Fig. 2:**
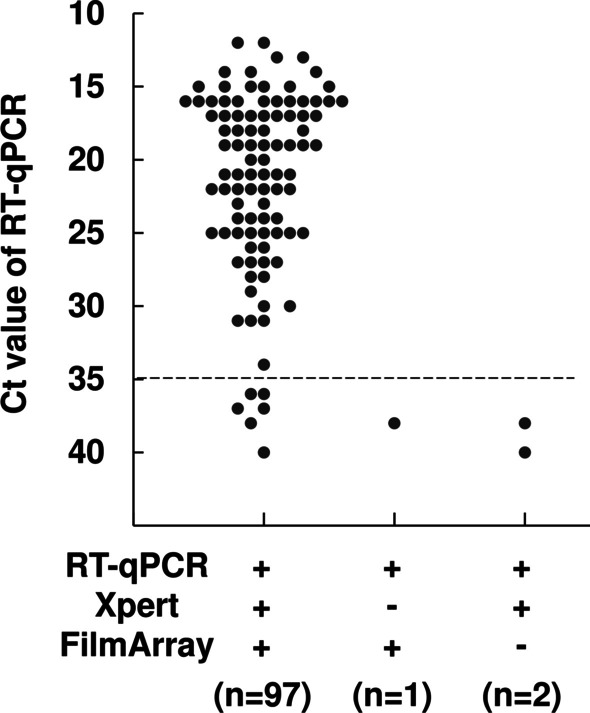
Relationship between Ct values and RT-qPCR results. Ninety-seven samples were positive for all tests (RT-qPCR, Xpert, and FilmArray), one sample was negative for Xpert only, and two samples were negative for FilmArray only. The dotted line indicates a Ct value of 35. + positive; − negative

To examine the detectable range of viral loads, the results of three PCR tests (RT-qPCR, Xpert, and FilmArray) were compared. There were 97 samples with positive results determined by all three tests (Fig. 2 and Table 1). Among these 97 positive samples with three tests, the mean viral load was 5.0 log_10_ copies/mL (0.2–8.0 log_10_ copies/mL), the mean RT-qPCR Ct value was 21.5 (range 12–40), the mean Xpert Ct value (N2) was 23.7 (range 14.2–42.6), and the mean Xpert Ct value (E) was 21.7 (range 12.6–42) (Table 1).

**Table 1:** Results of RT-qPCR, Xpert, and FilmArray

RT-qPCR	Xpert	FilmArray	Number of samples (n = 165)	Mean viral loads of RT-qPCR [log_10_ copies/mL] (range)*	Mean RT-qPCR Ct value (range)	Mean Xpert Ct value (N2) (range)	Mean Xpert Ct value (E) (range)
Pos	Pos	Pos	97	5.0 (0.2–8.0)	21.5 (12–40)	23.7 (14.2–42.6)	21.7 (12.6–42)
Pos	Pos	Neg	2	0.7 (0–1.4)	39 (38–40)	40.4 (39.1–41.6)	44.1*
Pos	Neg	Pos	1	0.8	38	NA	NA
Pos	Neg	Neg	0	NA	NA	NA	NA
Neg	Pos	Pos	0	NA	NA	NA	NA
Neg	Pos	Neg	0	NA	NA	NA	NA
Neg	Neg	Pos	0	NA	NA	NA	NA
Neg	Neg	Neg	65	NA	NA	NA	NA

*Pos* positive, *Neg* negative, *RT-qPCR* quantitative reverse transcriptase polymerase chain reaction, *Ct* threshold cycle, *N* nucleocapside, *E* envelope, *NA* not applicable

*One of the two samples did not exceed the threshold, and the Ct value could not be determined

Of 100 RT-qPCR positive samples, there were 91 samples with RT-qPCR Ct values < 35 and 9 samples with Ct values ≥ 35 (Fig. 2). Among 91 samples with Ct < 35, all samples were detectable with both Xpert and FilmArray (Fig. 2). Among nine samples with Ct ≥ 35, six samples were detected by both Xpert and FilmArray, one sample was not detected by Xpert (the viral load was 0.8 log_10_ copies/mL and the RT-qPCR Ct value was 38), and two samples were not detected by FilmArray (viral loads of 1.4 and 0 log_10_ copies/mL and RT-qPCR Ct values of 38 and 40, respectively) (Fig. 2 and Table 2). Therefore, all discordant samples had very low viral loads.

**Table 2:** Samples with discrepant test results

No	RT-qPCR	Xpert	FilmArray	Viral loads of RT-qPCR (log_10_ copies/mL)	Ct value of RT-qPCR	Ct value of Xpert (N2/E)
1	Pos	Neg	Pos	1.4	38	NA/NA
2	Pos	Pos	Neg	0.8	38	39.1/44.1
3	Pos	Pos	Neg	0	40	41.6/NA

*Pos* positive, *Neg* negative, *RT-qPCR* quantitative reverse transcriptase polymerase chain reaction, *Ct* threshold cycle, *N* nucleocapside, *E* envelope

*NA* not applicable

### Correlation between antigen level, Ct values and viral loads

**Fig. 3:**
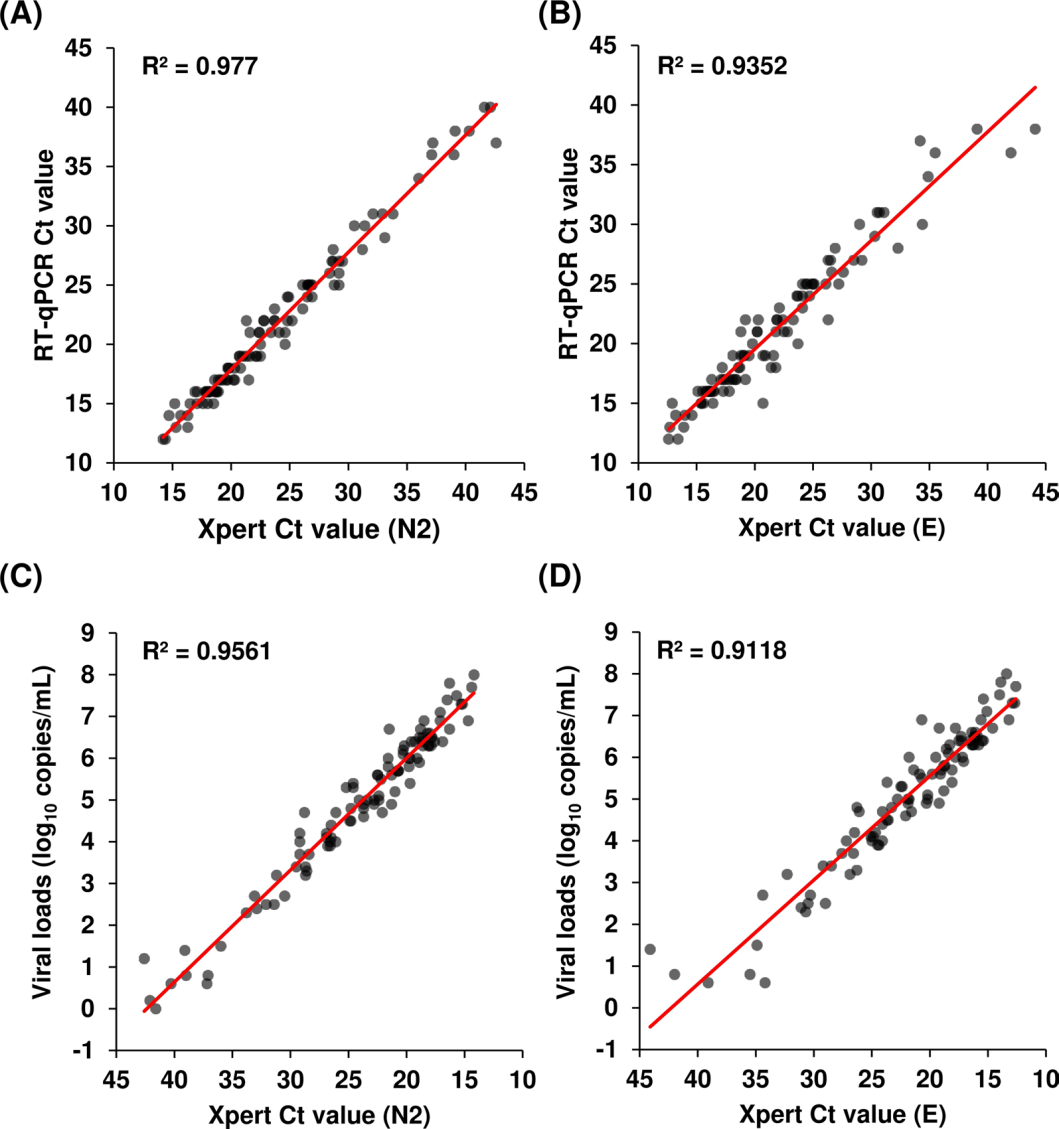
Correlation between the Ct value of RT-qPCR, viral load, and Ct value of Xpert. **A, B** Correlation between the Ct value of RT-qPCR and the Ct value of Xpert. **C**, **D** Correlation between the viral load determined by RT-qPCR and the Ct value of Xpert. Xpert was plotted for the Ct values of N2 (**A** and **C**) and E (**B** and **C**). The decision coefficient (R^2^) is shown in the scatter plot

The FilmArray is a qualitative test, whereas Xpert targets two different genes, including *N* and *E*, and yields Ct values. Thus, we examined the correlation between the Xpert Ct values, RT-qPCR Ct values, and viral loads. The results showed that the Xpert Ct values were highly correlated with the RT-qPCR Ct values for both N2 and E (Fig. 3A and B; R^2^ = 0.977, Xpert Ct (N2) vs RT-qPCR Ct; R^2^ = 0.935, Xpert Ct (E) vs RT-qPCR Ct). In addition, the viral load measured by RT-qPCR and the Xpert Ct value also showed a high correlation (Fig. 3C and D; R^2^ = 0.956, Xpert Ct (N2) vs viral load; R^2^ = 0.912, Xpert Ct (E) vs viral load). Because RT-qPCR amplifies the *N* gene, the data of Xpert N2 were shown to have a higher correlation than E. These results indicate that the Xpert Ct value accurately reflected the viral load in nasopharyngeal swabs.

**Fig. 4:**
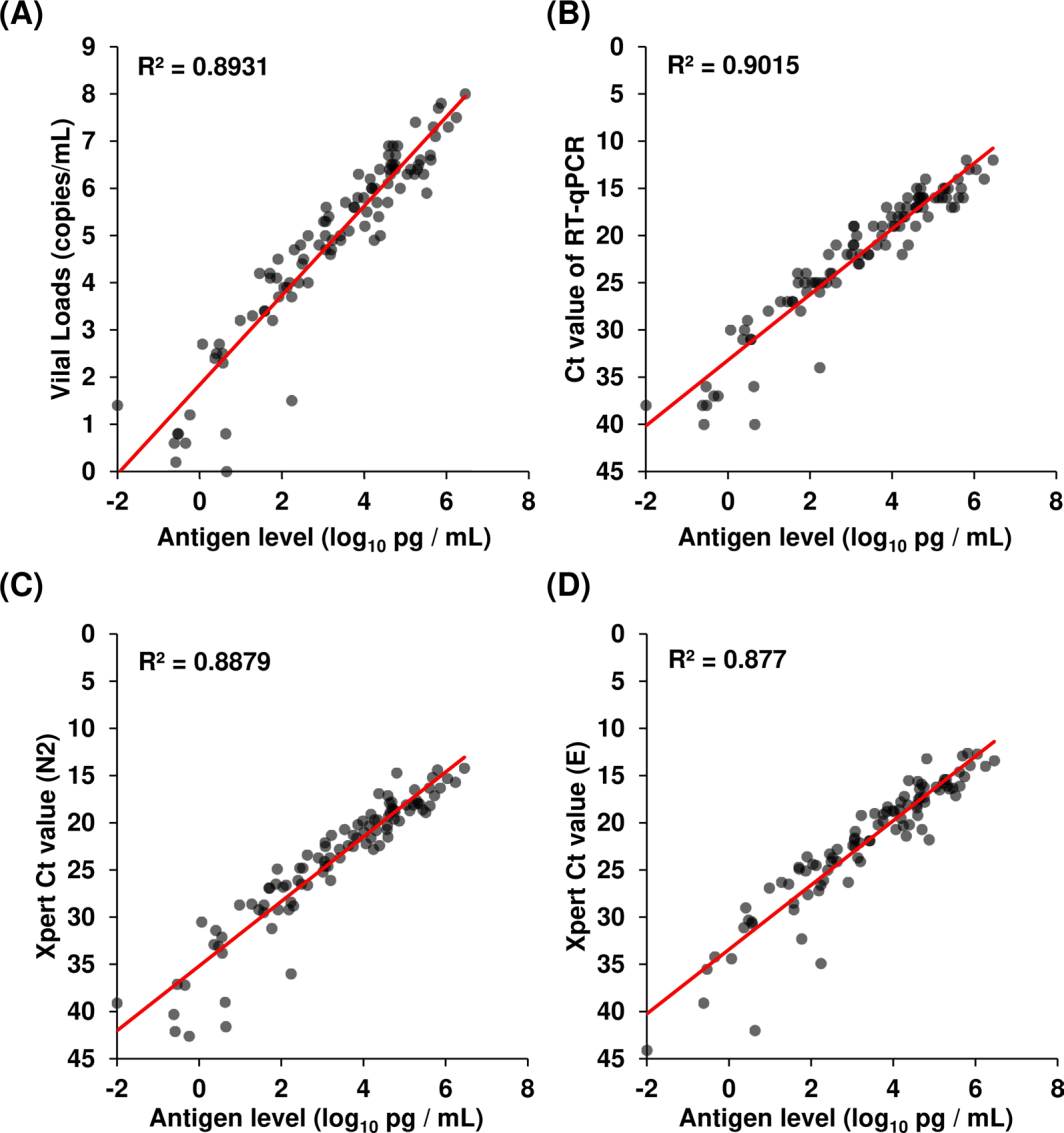
Correlation between the antigen level, viral load and Ct value. **A** Correlation between the antigen level and the viral load determined by RT-qPCR. **B** Correlation between the antigen level and the Ct value of RT-qPCR. **C**, **D** Correlation between the antigen level and the Ct value of Xpert. Xpert was plotted for the Ct values of N2 (**C**) and E (**D**). The decision coefficient (R^2^) is shown in the scatter plot

We also examined the correlation between the antigen levels, viral loads, RT-qPCR Ct values, and Xpert Ct values. As we previously reported [12], there was a correlation between antigen levels and viral loads (R^2^ = 0.893, Fig. 4A) or RT-qPCR Ct values (R^2^ = 0.902, Fig. 4B). Similarly, there was a correlation between the antigen level and the Xpert Ct values in N2 (R^2^ = 0.888, Fig. 4C) and E regions (R^2^ = 0.877, Fig. 4D).

## Discussion

We evaluated the performance of the newly installed Xpert system in our hospital. The results showed the test performance of Xpert was almost equivalent to that of FilmArray or RT-qPCR. Meanwhile, the Lumipulse antigen test showed a slightly lower detection rate than the NAAT (i.e., RT-qPCR, Xpert and FilmArray). In particular, the Lumipulse test judged an inconclusive result in a low viral load sample. In this case, the NATT test must be used to determine whether the sample is positive or negative. There was a high correlation between the Xpert Ct value and the RT-qPCR Ct value, viral load and antigen level. Of 165 samples, only one sample (Ct = 38) was discordant between Xpert and RT-qPCR, which was attributed to a very low viral load. Collectively, Xpert yielded accurate test results and quantitatively evaluated the viral load in samples [23]. Therefore, Xpert is considered to be a particularly useful method for random access SARS-CoV-2 testing.

Unlike the rapid antigen test (i.e., paper-based assays or lateral flow immunochromatography), the Lumipulse antigen test is based on the chemiluminescence enzyme immunoassay [10]. Compared with the assay performance of the rapid antigen test by other groups [24–29], our data show that the Lumipulse antigen test is relatively consistent with the NAAT analysis results among antigen tests [12, 30]. In addition, the Lumipulse antigen test specifically determined a positive result with a high accuracy in specimens with a viral load of more than 100 copies/mL [10]. Our previous analysis showed that NAAT detected the virus for a long time after the onset of symptom, while the Lumipulse antigen test becomes negative [11]. It was often observed that Lumipulse antigen tests turned to be negative after 10 days of symptom onset [12], which is consistent with the time when infectious viruses are detected in vitro [31–33].

The use of random access automated testing is removing barriers to conventional PCR test. The PCR test is the gold standard, but its process is time consuming because a certain number of samples are collected and analyzed in batches. In addition, it requires a skill, which limits its use by personnel who are not familiar with the PCR test. In contrast, Xpert, FilmArray, and Lumipulse take advantage of random access detection, which is useful for emergency. The turnaround time for Xpert, FilmArray, and Lumipulse is within 45 min, 45 min, and 35 min. In Xpert, if the amplification plot reaches the threshold, the test results can be returned in a shorter time. In addition, the simplicity of operation allows for testing to be performed in the absence of experienced personnel. Combining multiple testing methods will improve the speed and efficiency of the testing system in hospitals.

Using different tests that take advantage of on-demand nucleic acid amplification is an effective strategy. Xpert targets a small number of genes (often a single pathogen) due to the limited number of fluorescence wavelengths that can be detected and is inexpensive. In comparison, FilmArray amplifies several targets by multiplex PCR and then performs nested-PCR in multiple wells, which allows for a large number of target genes to be detected simultaneously, but it is more expensive. The Ct value and amplification curve can be confirmed using the Xpert system but not the FilmArray because it is a qualitative test. Therefore, the Xpert may be more advantageous for universal use in testing for SARS-CoV-2 in suspected patients, and the FilmArray may be more useful in patients with underlying medical conditions or children who are at risk of severe illness due to viral infections other than SARS-CoV-2.

In this study, we compared the Xpert Ct values with the RT-qPCR Ct values as a reference and found an extremely high correlation (Fig. 3A and B). The correlation was higher for Xpert (N2) than for Xpert (E), likely because RT-qPCR targets the *N* gene. However, the absolute Ct values of RT-qPCR were closer to those of Xpert (E) than to those of Xpert (N2), except for samples with low viral loads (Tables 1 and 2). In some samples with low viral loads, the Xpert Ct (N2), but not the Xpert Ct (E), could be measured as previously reported [23].

The Ct value is useful as a surrogate indicator of viral load by RT-PCR. However, it should be carefully compared with other datasets because the Ct value changes depending on the following factors. First, it is depended on the target region of PCR amplification. In particular, in the case of in-house assays, it is necessary to carefully consider whether the same conditions are used for primer sequences, reagents, and measuring equipment. Next, the Ct value varies depending on the threshold setting. It is necessary to make sure that the threshold of the assay used is always at a certain level. Therefore, if the amount of virus is to be examined accurately, it is better to measure a standard sample and measure viral load quantitatively from the calibration curve.

Overall, the Xpert, FilmArray, and Lumipulse tests could detect SARS-CoV-2 in samples with sufficient viral loads. The only inconsistency was observed in specimens with low viral loads, but these patients likely release less live virus [31, 32, 34]. In fact, we previously demonstrated that discrepant results between the Lumipulse antigen test and RT-qPCR were attributable to low viral loads collected from seropositive patients [12]. Therefore, the use of random access detection may be effective for patient triage by enabling the rapid return of test results.

## Conclusions

In this study, we evaluated the performance of two NAATs (Xpert and FilmArray) and a quantitative antigen test (Lumipulse) to detect SARS-CoV-2. When the result of RT-qPCR was used as reference, the test results of Xpert and FilmArray showed high agreement, indicating that these tests have almost equivalent test accuracy. The Lumipulse antigen test had a slightly lower concordance rate than Xpert and FilmArray, but had high test accuracy except for samples with low viral load. Xpert Ct value correlated with RT-qPCR Ct value, viral load and antigen level, helping to understand the phases of infection. Xpert, FilmArray and Lumipulse tests can be performed automatically on dedicated equipment, achieving seamless and accurate testing for SARS-CoV-2 without the need for complicated procedures.

## Data Availability

The datasets used and/or analysed during the current study are available from the corresponding author on reasonable request.
